# ZFAS1/STAT3 axis modulates imatinib resistance of chronic myeloid leukemia cells through glucose metabolism reprogramming

**DOI:** 10.3389/fonc.2025.1603060

**Published:** 2025-07-08

**Authors:** Lan Yang, Yanqiu Han

**Affiliations:** Department of Hematology, Affiliated Hospital of Inner Mongolia Medical University, Hohhot, Inner Mongolia, China

**Keywords:** chronic myeloid leukemia, imatinib resistance, ZFAS1, stat3, glucose metabolism reprogramming

## Abstract

**Background:**

Chronic myeloid leukemia (CML) is a myeloproliferative neoplasm characterized by the presence of the Philadelphia chromosome (chromosome 22). This cytogenetic abnormality gives rise to the *BCR::ABL1* fusion gene, which encodes the constitutively active BCR-ABL1 protein tyrosine kinase, driving uncontrolled proliferation and impaired apoptosis of hematopoietic stem and progenitor cells, leading to leukemogenesis. Imatinib mesylate (IM), a first-generation tyrosine kinase inhibitor (TKI) specifically targeting the BCR-ABL1 oncoprotein, represents the standard first-line therapy for patients with CML. However, imatinib resistance remains a major therapeutic challenge.

**Objective:**

This study aims to elucidate the role of the *ZFAS1/STAT3* signaling axis in mediating imatinib resistance in CML by promoting metabolic reprogramming, with a particular focus on alterations in glucose metabolism.

**Methods:**

Imatinib-resistant (IM-R) K562 cells were used to investigate the functional role of *ZFAS1*gene. Following *ZFAS1* knockdown, assessments of cell viability, apoptosis, and glucose metabolism were performed. The interaction between ZFAS1 and IGF2BP2, as well as its regulatory effect on *STAT3* expression and glycolysis-related genes (including *HIF1α*, *LDHA*, and *PDK1*) were examined using qRT-PCR and western blotting. Additionally, the impact of STAT3 overexpression and glycolysis inhibition (2-DG) on IM sensitivity were examined.

**Results:**

Our findings revealed that *ZFAS1* expression was significantly upregulated in IM-R CML patient samples and IM-R K562 cells. Silencing of *ZFAS1* enhanced cellular sensitivity to IM, inhibited glucose metabolism reprogramming, and promoted apoptosis. Mechanistically, *ZFAS1* was found to interact with IGF2BP2, facilitating the stabilization of *STAT3* mRNA and leading to increased *STAT3* expression. This, in turn, resulted in the upregulation of key glycolytic genes. Overexpression of *STAT3* reversed the effects of *ZFAS1* knockdown by restoring glycolytic activity and re-establishing IM resistance. Additionally, 2-DG treatment effectively reversed STAT3-induced IM resistance by inhibiting glycolysis.

**Conclusion:**

These findings demonstrate that the ZFAS1/STAT3 signaling axis contributes to imatinib resistance in CML through the modulation of glucose metabolism. Targeting this regulatory pathway may represent a novel therapeutic strategy to overcome TKI resistance in CML.

## 1 Introduction

Chronic myeloid leukemia (CML) is a clonal hematopoietic stem cell malignancy characterized by the presence of the Philadelphia chromosome. This chromosomal abnormality facilitates the production of the *BCR::ABL1* fusion gene (1, 2). This genetic alteration results in the production of an oncoprotein, BCR-ABL1, with abnormally high tyrosine kinase activity, promoting the uncontrolled growth of white blood cells (2). Tyrosine kinase inhibitors (TKIs), represented by imatinib mesylate (IM), are the first-line drugs for the treatment of CML, which specifically block the binding site of ATP on Abl kinase. Despite considerable progress in the treatment of CML, resistance to these targeted therapies continues to pose a significant obstacle (3, 4). Compared with traditional treatment drugs, such as interferon, busulfan, hydroxyurea and so on, IM notably enhances treatment efficacy and early survival rates in patients with CML (5). Nonetheless, imatinib does not provide a definitive cure for CML, and resistance to imatinib may develop over time. In patients with advanced-stage CML, the therapeutic efficacy of imatinib is often limited, and it frequently fails to prevent disease relapse or progression. Moreover, the emergence of imatinib resistance represents a major clinical challenge, significantly undermining the long-term effectiveness of this targeted therapy.

The mechanisms underlying imatinib resistance in CML are complex as two categories: *BCR-ABL1*-dependent and *BCR-ABL1*-independent *(* 6). The emerging mechanisms of imatinib resistance in CML is the dysregulation of long non-coding RNAs (lncRNAs) (7). LncRNAs have emerged as critical modulators of gene expression and cellular signaling. Among these, ZFAS1, a well-characterized oncogenic lncRNA, plays a crucial role in the progression and resistance of various cancerous types, but its mechanistic role in CML remains underexplored (8, 9). In parallel, the signal transducer and activator of transcription 3 (STAT3) signaling pathway has been implicated in the emergence of drug resistance in CML due to its regulatory influence on various cellular processes, including proliferation, survival, and metabolism (10, 11). Previous study has shown that *BCR-ABL1*, a hallmark of CML, activates STAT3 via both the Janus Kinase (JAK) and mitogen-activated protein kinase/extracellular signal-regulated kinase (MEK/ERK) pathways (12). Specifically, BCR-ABL1 phosphorylation of STAT3 is implicated in promoting CML progression and resistance to treatment. Also, *ZFAS1* inhibits the progression of triple-negative breast cancer by negatively regulating the *STAT3* gene (13). This inhibitory effect of *ZFAS1* on *STAT3* has also been observed in gastric and non-small cell lung cancers (14, 15). Furthermore, *STAT3* mediated regulation of glucose metabolism in types of cancer (16–19).

Recent evidence indicates that glucose metabolism reprogramming, a hallmark of cancer, has also been associated with the development of treatment resistance in CML and other malignancies (20, 21). Compared to other metabolic pathways such as lipid or amino acid metabolism, glucose metabolism is more directly implicated in supporting the high energy demands of CML cells and their resistance to tyrosine kinase inhibitors (22–25). However, the upstream lncRNA-based regulatory mechanisms governing this glycolytic shift in CML remain poorly defined. This gap in knowledge prompts further investigation into the potential link between *ZFAS1*, *STAT3*, and glucose metabolism, with the goal of elucidating novel mechanisms of metabolic reprogramming in CML. Given the central role of *STAT3* in regulating metabolic pathways in various cancers and its role with *ZFAS1* mentioned above, we hypothesize that *ZFAS1* may modulate *STAT3* activation or function, thereby contributing to imatinib resistance in CML through the reprogramming of glucose metabolism. In this study, we aim to investigate the role of the *ZFAS1/STAT3* axis in promoting imatinib resistance in CML through the regulation of glucose metabolism pathway. Elucidating these mechanisms may provide valuable insights into the pathogenesis of drug resistance in CML and aid in the development of novel therapeutic strategies to enhance treatment efficacy.

By elucidating the mechanisms by which *ZFAS1* and *STAT3* contribute to imatinib resistance, our study offers new insights into the metabolic adaptations of CML cells. These findings advance our understanding of CML pathophysiology and establish a foundation for the development of innovative therapeutic approaches targeting the *ZFAS1/STAT3* axis. Such strategies hold promise for overcoming imatinib resistance and ultimately improving clinical outcomes in patients with CML.

## 2 Methods

### 2.1 Ethics approval and consent to participate

This study was approved by the Ethics Committee of Inner Mongolia Medical University (Inner Mongolia, China; Approval number: No. YKD202302030). All patients provided written informed consent to participate in the study. The clinical samples were collected between June 2023 and January 2024, and all patient data were anonymized to ensure confidentiality. Ethical approval for the study was obtained in accordance with the Declaration of Helsinki. Chinese patients with chronic-phase CML (Philadelphia chromosome-positive), who had been receiving imatinib (400 mg/day) as first-line therapy, were selected for this study. Molecular responses were monitored at 3, 6, and 12 months post-treatment using quantitative PCR for the *BCR::ABL1* fusion gene. Patients who failed to achieve a major molecular response (MMR) or complete molecular response (CM-R, n = 30), which defined as a *BCR::ABL1/ABL1* ratio ≥1%—by 12 months were considered imatinib-resistant (IM-R) and subsequently switched to second-generation tyrosine kinase inhibitors (dasatinib or nilotinib). Patients who achieved CMR or MMR were categorized as imatinib-sensitive (IM-S, n = 30).

### 2.2 Bioinformatics analysis

LncATLAS database (https://lncatlas.crg.eu/?tdsourcetag=s_pcqq_aiomsg) was used to predict *ZFAS1* location in cells. Using StarBase database (https://rnasysu.com/encori/index.php), we investigated the potential regulatory relationship between *ZFAS1* and *STAT3* expression through IGF2BP2. To identify potential RNA-binding proteins (RBPs) mediating the interaction between ZFAS1 and STAT3, the StarBase database was utilized. First, the “RBP-Target” module was accessed, and the RBP–lncRNA analysis function was employed by inputting “ZFAS1” to screen for RBPs predicted to interact with this lncRNA. Subsequently, the RBP–RNA module was queried using “STAT3” to identify RBPs that bind to the STAT3 transcript. IGF2BP2 emerged as a common interacting protein for both ZFAS1 and STAT3. IGF2BP2 was selected for further investigation based on existing literature indicating its regulatory role in glycolysis within myeloid leukemia cells (26).

### 2.3 Cell culture

Human leukemic cell line K562 cells were procured from ATCC in Manassas, VA, USA, and maintained in RMPI 1640 medium supplemented with 10% heat-inactivated FBS, 100 U/ml penicillin, and 100 μg/ml streptomycin. Cultures were incubated at 37°C in a humidified atmosphere containing 5% CO_2_. IM, sourced from Sigma (MO, USA), was generated imatinib-resistant K562 cells, employing a previously described method (27, 28).

### 2.4 Transfection and treatment

The siRNA targeting *ZFAS1* (si-ZFAS1) and the negative control siRNA (si-NC) were purchased from Thermo Fisher Scientific Co., Ltd. (USA). The *ZFAS1* reference sequence (accession number: NR_003604.3) was retrieved from the NCBI. The sequences of *ZFAS1* siRNA were as follows: forward, 5’-GAUGAUCUAUGGAAUUUCATT-3’ and antisense: 5’-UGAAAUUCCAUAGAUCAUCTT-3′. The sequence of the negative control siRNA (si-NC) used in this study is: Forward, 5’-UUCUUCCGAACGUGUCACGUTT-3’ and antisense: 5’-ACGUGACACGUUCGGAGAATT -3′. Using Lipofectamine^®^ 2000 (Invitrogen) in accordance with the manufacturer’s protocol, siRNA was transfected into the K562 cell lines. To study the function of *STAT3*, we transfected the *STAT3* plasmid into IM-R CML cells using Lipofectamine 2000 reagent (Thermo Fisher Scientific, USA). Empty plasmid serves as negative control (NC). First, cells were seeded in culture plates at a density of 2×10^5^ cells/mL and cultured for 24 hours in antibiotic-free medium to allow recovery prior to transfection. Subsequently, Lipofectamine 2000 was used to prepare the transfection complex according to the manufacturer’s instructions. The *STAT3* plasmid (2 µg) was mixed with the transfection reagent in 200 µL of Opti-MEM medium, left to stand for 20 minutes, and then added to the cell culture medium. After transfection, the cells were further cultured for 48 hours in a 37°C, 5% CO_2_ incubator. After a 48-hour transfection period, the cells were harvested and utilized in subsequent assays. The transfection efficiency was assessed by detecting the expression level using qRT-PCR.

### 2.5 Cytotoxicity assay

Cell counting kit-8 (CCK-8) kit was used to assess cytotoxicity under various treatment conditions. To detect drug sensitivity, imatinib-resistant K562 (IM-R K562) cells, with or without treatment with 2 mM of the glycolysis inhibitor 2-Deoxy-D-glucose (2-DG; 154-17-6, Ann Arbor, MI, USA), and imatinib-resistant K562 cells transfected with si-NC, si-ZFAS1, NC, or *STAT3* plasmid, were seeded in a 96-well plate at a density of 2,000 cells per well, with medium containing different concentrations of IM. A concentration of 2 mM 2-DG was used in this study based on previous report indicating effective glycolysis inhibition in leukemia cells (22). Each concentration was tested in triplicate. After 48 hours of incubation, 10 μL of CCK-8 solution (Boster Bio, Wuhan) was added to each well. Two hours later, optical density (OD) measurements for cells in each group were then performed using a microplate reader at a wavelength of 450 nm. Drug resistance was assessed by comparing the IC50 values (the drug concentration required to inhibit cell growth by 50%) derived from the growth inhibition curves.

### 2.6 Colony formation assay

A colony formation assay was conducted to evaluate the ability of IM-R K562 cells, transfected with either si-NC or si-ZFAS1, to form colonies after a 14-day IM treatment. IM-R K562 cells were transfected with si-NC and si-ZFAS1 as needed. Single-cell suspensions were prepared and quantified. The cells were cultured in medium containing 2 μM IM, with fresh IM-supplemented medium replacing the existing medium every 3–4 days. After 14 days, colony formation was assessed, with a colony defined as a cluster of at least 50 cells. The number of colonies per well was counted manually or using an automated colony counter. Colony formation efficiencies of the si-NC and si-ZFAS1 transfected groups under IM treatment were compared. Efficiency was calculated by dividing the number of colonies by the initial cell inoculation number and multiplying by 100. The impact of *ZFAS1* knockdown on the colony-forming ability of IM-R K562 cells under imatinib treatment was subsequently analyzed.

### 2.7 Flow cytometry analysis for apoptosis detection

Cells were incubated in a 5% CO_2_ incubator at 37°C for 48 hours. After 48 h, cells were collected by centrifugation at 300 g for 5 min. Cells were washed twice with cold PBS and re-suspended at a concentration of 1 × 10^6^ cells/mL in 1X binding buffer. A total of 100 μL of cell suspension (1 × 10^5^ cells) was divided into 5 mL culture tubes. Each tube was supplemented with 5 µL of Annexin V-FITC and 5 µL of propidium iodide (PI). Following incubation, 400 µL of 1 × binding buffer was added to each tube. Proper compensatory controls and gating strategies were implemented to accurately distinguish between viable cells. Detection of si-NC group and si-ZFAS1 apoptosis cell percentage (Annexin V positive). The effect of *ZFAS1* knockdown on IM-R K562 cell apoptosis induced by imatinib was evaluated by comparing the apoptosis rate of the two group. Flow cytometry was performed using a BD FACSCalibur system (BD Biosciences). At least 10,000 events were acquired per sample. Data were analyzed using FlowJo v10 software. Viable cells were gated using forward scatter (FSC) and side scatter (SSC), and apoptotic cells were defined as Annexin V-FITC positive.

### 2.8 Quantitative real-time polymerase chain reaction assay

Total RNA was isolated from peripheral blood samples from CML patients or cultured K562 cells following treatment with IM. The RNA samples were dissolved in RNase-free distilled water. Subsequently, 2 μg of total RNA was reverse transcribed into cDNA using the High-Capacity RNA to cDNA Kit (ThermoFisher Scientific, Shanghai, China). Quantitative PCR analysis of gene expression was conducted in 96-well plates, with each well containing a 20 µl reaction mixture consisting of 2 × SYBR green master mix (NZYtech, Portugal), diluted gene-specific primers, and cDNA. qPCR data were analyzed using QuantStudio Design & Analysis software v1.5. The primers were sourced from OriGene Technologies, Inc., and their sequences are detailed in **Table 1**. Relative gene expression was calculated using the 2^-ΔΔCt^ method with *GAPDH* or *U6* as the internal control.

**Table 1:** The primer sequences used in quantitative PCR assay.

Gene name	Forward 5′-3′	Reverse 5′-3′
*ZFAS1*	ACGTGCAGACATCTACAACCT	TACTTCCAACACCCGCAT
*STAT3*	CTTTGAGACCGAGGTGTATCACC	GGTCAGCATGTTGTACCACAGG
*GAPDH*	GTCTCCTCTGACTTCAACAGCG	ACCACCCTGTTGCTGTAGCCAA
*U6*	CGCTTCGGCAGCACATATAC	TTCACGAATTTGCGTGTCATC

### 2.9 Cytoplasmic/nuclear fractionation

For the cytoplasmic/nuclear separation experiment, cells were cultured, harvested, and were lysed to separate the cytoplasmic and nuclear fractions by Nuclear and Cytoplasmic Extraction Reagents (78833, Thermo Fisher) according to the manufacturer’s instructions. The cytoplasmic and nuclear RNA was then isolated according to the recommended protocols, followed by reverse transcription and qRT-PCR.

### 2.10 RNA fluorescence *in situ* hybridization

RNA FISH was conducted using the Fluorescent *in situ* Hybridization Kit (lnc1CM001, RiboBio) following the manufacturer’s protocol. Cy3-labeled probes targeting *ZFAS1* were synthesized by RiboBio (Guangzhou, China). Fluorescent signals were captured using a confocal laser scanning microscope (FV3000, Olympus).

### 2.11 Western blot assay

For Western blot assays, K562 cells were seeded at a density of 1 × 10^6^ cells/mL in T25 flasks and incubated for 48 hours after transfection or treatment. Approximately 5 × 10^6^ cells per sample were harvested, lysed in RIPA buffer supplemented with protease and phosphatase inhibitors. The Bradford Protein Assay Kit (Boster Bio, Wuhan, China) was employed to determine protein concentration. Afterward, PVDF membranes post-incubation with primary antibodies were exposed to goat anti-rabbit IgG (1:2000, PR30011, Proteintech) as a secondary antibody. Protein bands were visualized using an enhanced chemiluminescence (ECL) system (Millipore), and band intensities were quantified using Image Lab software (BioRad, US). The primary antibodies, which included LDHA (1:500, Cat No. # PA5-27406), HIF-1α (1:2000, Cat No. #MA1-516), PDK1 (1:5000, Cat No. #MA5-32702), STAT3 (1:500, Cat No. #MA5-157126) and GAPDH (1:1000, Cat No. #MA5-15738), were acquired from Thermo Fisher ^®^ (Shanghai, China).

### 2.12 RNA immunoprecipitation assay

To examine the interaction between *ZFAS1* and IGF2BP2 in the context of gene silencing, IM-R K562 cells were first transfected with si-NC or si-ZFAS1 using Lipofectamine 2000 according to the manufacturer’s protocol. After 48 hours of transfection, approximately 1 × 10^7^ cells per sample were harvested and lysed for RIP assays. RIP was performed using the Magna RIP RNA-Binding Protein Immunoprecipitation Kit (Millipore, USA). Antibodies targeting IGF2BP2 (Proteintech, Cat No.11601-1-AP) were conjugated to protein A/G magnetic beads to capture complexes involving IGF2BP2. Subsequently, the cell lysate was mixed with magnetic beads conjugated with specific antibodies, promoting the formation of complexes between IGF2BP2 and its interacting partners. Thorough washing of the beads was performed to eliminate non-specifically bound proteins and contaminants, ensuring the specificity of the captured interactions. The complexes bound to the beads were then eluted, releasing the proteins associated with IGF2BP2, including any enriched *STAT3* molecules. The data obtained from the RIP assay were analyzed to evaluate the extent of *STAT3* enrichment by IGF2BP2, shedding light on their potential interaction and functional significance.

### 2.13 mRNA stability assay

CML cells were treated with actinomycin D (5 μg/mL) to inhibit transcription. RNA was harvested at 0, 2, 4, 8, 12 and 16 hours post-treatment. *STAT3* mRNA levels were quantified using qRT-PCR and normalized to *GAPDH*. The relative remaining mRNA percentage was calculated against the 0 h time point.

### 2.14 Oxygen consumption rate measurement and ECAR

The OCR was measured using the Oxygen Consumption Rate Assay Kit from Cayman Chemical (US). Prior to commencing the experiment, the existing culture medium was replaced with 160 µL of fresh medium supplemented with 10 µL of a phosphorescent oxygen probe solution. To prevent evaporation, 100μL of mineral oil was gently added to each well. Fluorescence signals were detected using a plate reader configured with excitation and emission wavelengths of 380 nm and 650 nm, respectively, and maintained at 37 °C for a duration of two hours. OCR values were normalized to the total cell count. The ECAR was simultaneously measured using the Seahorse XF Analyzer (Agilent Technologies). To ensure adherence of K562 suspension cells to the Seahorse XF96 plate, wells were pre-coated with Cell-Tak cell adhesive (Corning) according to the manufacturer’s protocol. Cells were then seeded and gently centrifuged at 200g for 1 minute to promote attachment before measurement. The ECAR measurements were carried out, and data were normalized to cell numbers to account for any variations in cell density.

### 2.15 Statistical analysis

Statistical analyses were performed using GraphPad Prism 9.0 (GraphPad Software Inc., San Diego, CA, USA). Normality of data was assessed using the Shapiro–Wilk test. For comparisons between two groups, unpaired two-tailed Student’s t-tests were used. For comparisons among multiple groups, one-way ANOVA followed by Tukey’s *post hoc* test was applied. All experiments were independently repeated at least three times, and data are presented as mean ± standard deviation (SD). A p-value of <0.05 was considered statistically significant.

## 3 Results

### 3.1 Elevated expression of LncRNA ZFAS1 and STAT3 in IM-R CML and K562 cells

**Figure 1:**
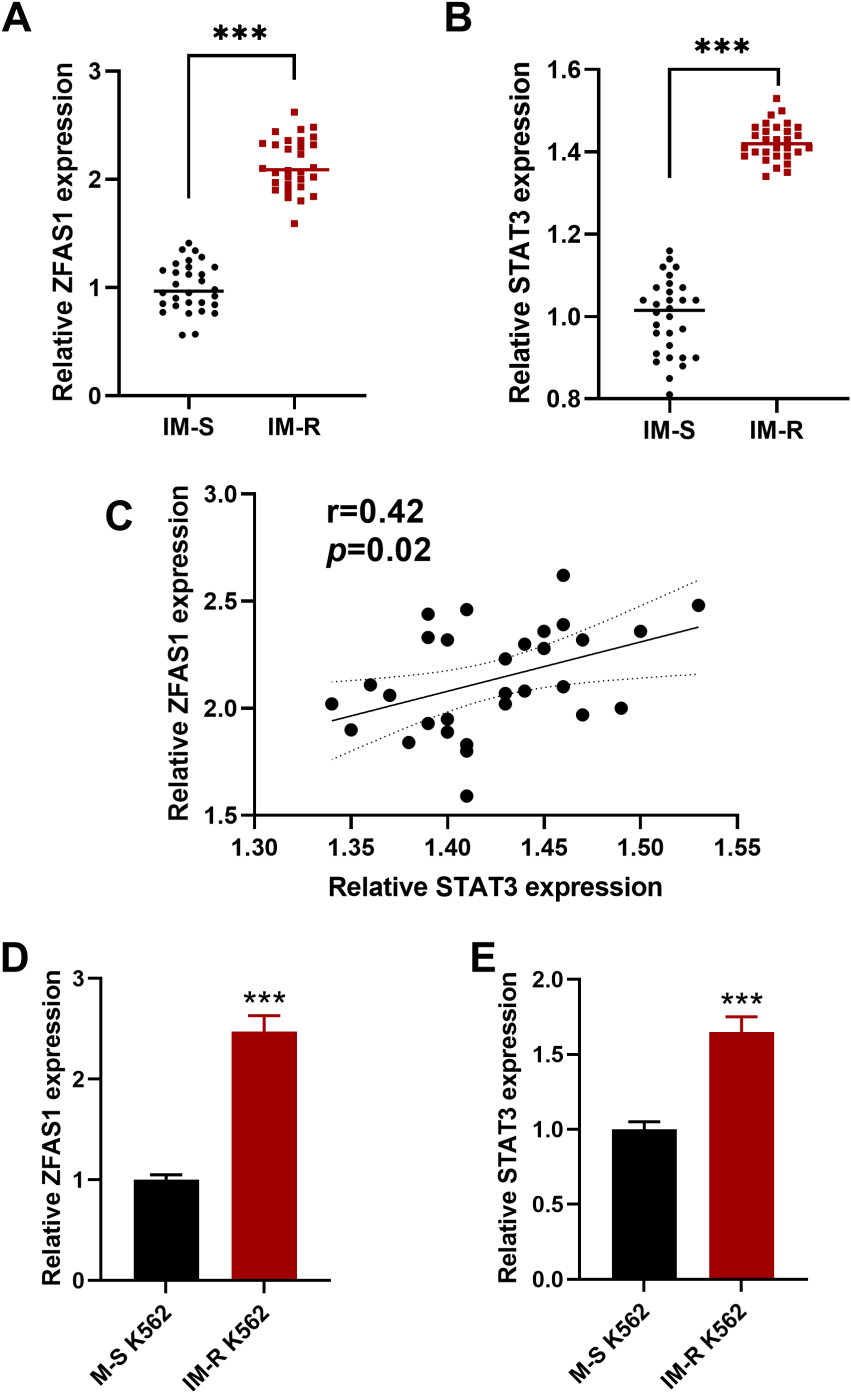
Elevated expression of lncRNA *ZFAS1* and *STAT3* in imatinib-resistant (IM-R) CML patient samples and IM-R K562 cell line. **(A, B)** Relative expressions of *ZFAS1* and *STAT3* in peripheral blood cells from imatinib-resistant (IM-R, n=30) and imatinib-sensitive (IM-S, n=30) CML patients. **(C)** Correlation analysis between *ZFAS1* and *STAT3* in CML patients samples. **(D, E)** Expression levels of lncRNA *ZFAS1* and *STAT3* in IM-R and IM-S K562 cells. Data are presented as mean ± SD. ***p<0.001 *vs*. IM-S.

We first assessed the expression levels of ZFAS1 and STAT3 in peripheral blood cells from CML patients. The IM-R group exhibited a significantly elevated expression of *ZFAS1* (2.14 ± 0.25) and *STAT3* (1.43 ± 0.05) compared to *ZFAS1* (1.00 ± 0.23) and *STAT3* (1.00 ± 0.09) in the imatinib-sensitive group (*p* < 0.001; **Figures 1A, B**). Further correlation analysis revealed a positive correlation between *ZFAS1* and *STAT3* in CML patients (r=0.4, *p*=0.02, **Figure 1C**). We then evaluated the expression of *ZFAS1* and *STAT3* in IM-R and IM-S K562 cell lines, and similarly observed significantly elevated levels in the IM-R K562 cells (2.47 ± 0.16) as compared to IM-S K562 cells (1.00 ± 0.05, *p* < 0.001; **Figures 1D, E**). These findings support the involvement of the *ZFAS1/STAT3* axis in both patient samples and *in vitro* models of imatinib resistance.

### 3.2 ZFAS1 knockdown increases sensitivity to imatinib and inhibits glucose metabolism reprogramming in IM-R K562 cells

**Figure 2:**
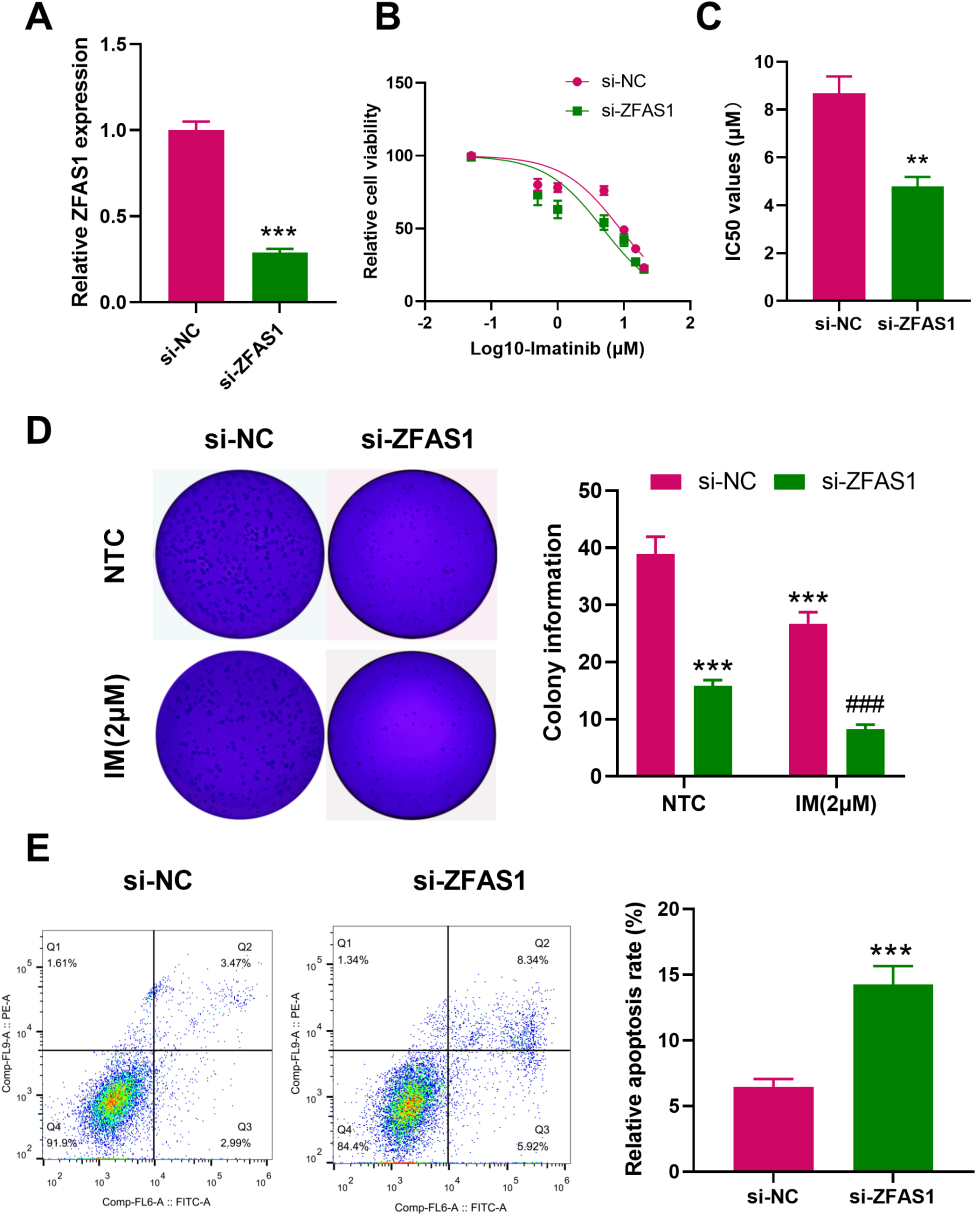
Effects of ZFAS1 knockdown on imatinib-resistant K562 cells. **(A)** PCR analysis showing the efficiency of ZFAS1 knockdown in IM-R K562 cells. **(B)** Drug sensitivity assay (CCK-8) showing relative cell viability of si-NC and si-ZFAS1 IM-R K562 cells treated with varying concentrations of IM. **(C)** IC_50_ values of imatinib in si-ZFAS1 cells and si-NC cells. **(D)** Colony formation assay showing the number of colonies formed by si-NC and si-ZFAS1 IM-R K562 cells treated with or without 2 µM IM. **(E)** Flow cytometry analysis showing the apoptosis rate of si-NC and si-ZFAS1 IM-R K562 cells treated with 2 µM IM. Data are presented as mean ± SD. NTC: non-treated control; **p<0.01, ***p<0.001 *vs*. si-NC; ^###^ *p*<0.001 *vs*. IM+si-NC.

After ZFAS1 knockdown, its expression level significantly decreased in IM-R K562 cells (0.29 ± 0.02) compared with the si-NC group (1.00 ± 0.05, *p <*0.001, **Figure 2A**). Drug sensitivity assays utilizing CCK-8 demonstrated that *ZFAS1* silencing significantly reduced cell viability and decreased the IC_50_ value of imatinib from 8.691 µM to 4.785 µM, indicating enhanced drug sensitivity in the si-ZFAS1 group compared to the si-NC group (**Figures 2B, C**). Colony formation assays further showed that *ZFAS1* knockdown impaired the proliferative capacity of K562 cells, independent of imatinib treatment. In the absence of imatinib, ZFAS1-silenced cells formed significantly fewer colonies (15.87 ± 1.00) were than the si-NC group (38.93 ± 3.00, *p <*0.001, **Figure 2D**), indicating that *ZFAS1* contributes to baseline cell proliferation. This inhibitory effect was further amplified upon treatment with 2 µM imatinib, with colony numbers reduced to 8.27 ± 0.80 in the si-ZFAS1 group compared to 26.73 ± 2.00 in the si-NC group (*p <*0.001, **Figure 2D**). Although the IM+si-NC group also exhibited a reduction in colony formation, this was attributed to the additive effect of imatinib and control siRNA transfection, rather than the transfection process itself. Therefore, the increased IM sensitivity is primarily attributable to the *ZFAS1* knockdown (**Figure 2D**). Knockdown of *ZFAS1* elevated the apoptosis rate in IM-treated (2 µM) imatinib-resistant K562 (IM-R K562) cells from 6.13% in the control group to 13.6% in ZFAS1-silenced cells (*p <*0.001, **Figure 2E**). Collectively, these findings suggest that *ZFAS1* knockdown enhances the sensitivity of IM-R K562 cells to imatinib, inhibits their proliferation, and promotes apoptosis.

**Figure 3:**
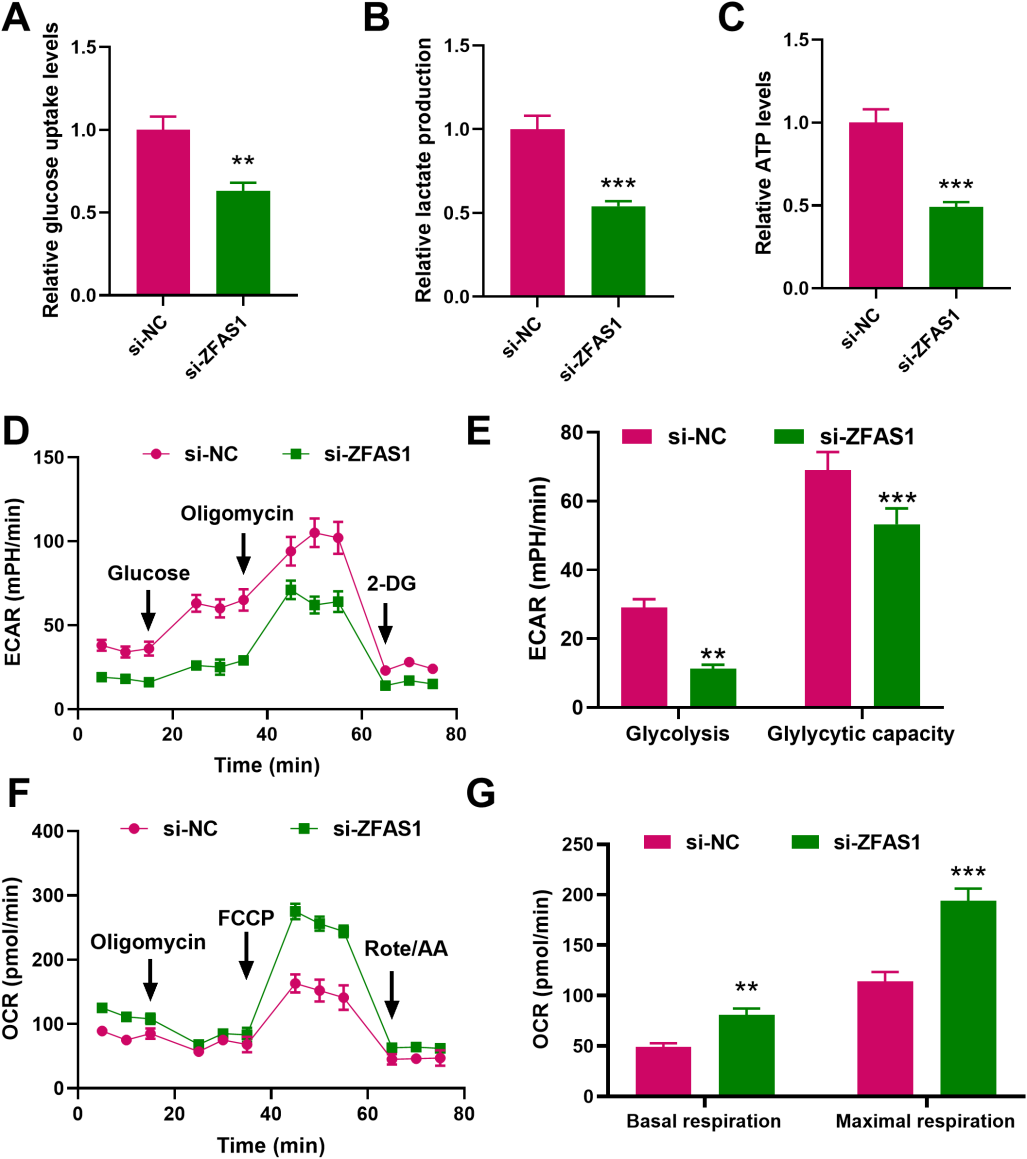
Effects of *ZFAS1* knockdown on glucose metabolism in IM-R K562 cells. **(A)** Relative glucose uptake levels in si-NC and si-ZFAS1 IM-R K562 cells. **(B)** Relative lactate production in si-NC and si-ZFAS1 IM-R K562 cells. **(C)** Relative ATP levels in si-NC and si-ZFAS1 IM-R K562 cells. **(D)** Extracellular acidification rate (ECAR) in si-NC and si-ZFAS1 IM-R K562 cells. **(E)** Oxygen consumption rate (OCR) in si-NC and si-ZFAS1 IM-R K562 cells. **(F)** ECAR *vs*. OCR plot illustrating the metabolic shift in si-ZFAS1 IM-R K562 cells. **(G)** Comprehensive metabolic profile showing the impact of *ZFAS1* knockdown on IM-R K562 cell metabolism. Data are presented as mean ± SD. ^**^ *p*<0.01, ^***^ *p*<0.001 *vs*. si-NC.

Knockdown of ZFAS1 significantly reduced glycolytic function in IM-R cells. Compared with the si-NC group, glucose uptake decreased from 1.00 ± 0.08 to 0.63 ± 0.05 (*p* < 0.001), lactate production decreased from 1.00 ± 0.08 to 0.54 ± 0.03 (*p* < 0.001), and ATP levels dropped from 1.00 ± 0.08 to 0.49 ± 0.03 (*p* < 0.001, **Figures 3A–C**). To further assess glycolytic function, ECAR was measured. As shown in **Figures 3D, E**, si-ZFAS1 cells exhibited a marked decrease in both basal glycolysis (11.33 ± 1.10, *p <*0.01) and glycolytic capacity (53.33 ± 4.6, *p <*0.01) compared to si-NC cells (29.52 ± 3.55; 69.54 ± 5.32). In contrast, mitochondrial oxidative phosphorylation was enhanced upon ZFAS1 knockdown. OCR analysis revealed that both basal (81 ± 6.2, *p <*0.01) and maximal respiration (194 ± 12.1, *p <*0.01) were significantly increased in si-ZFAS1 cells (49 ± 3.8; 114 ± 9.5, **Figures 3F, G**). These data suggested *ZFAS1* plays a crucial role in the regulation of a metabolic shift from glycolysis to oxidative phosphorylation in IM-R K562 cells. The inhibition of glucose uptake and glycolysis, coupled with increased oxidative phosphorylation, highlights the metabolic reprogramming induced by *ZFAS1* knockdown, which may contribute to the increased sensitivity to imatinib observed in these cells.

### 3.3 Mechanism of ZFAS1 regulation of glucose metabolism reprogramming through STAT3 and IGF2BP2

**Figure 4:**
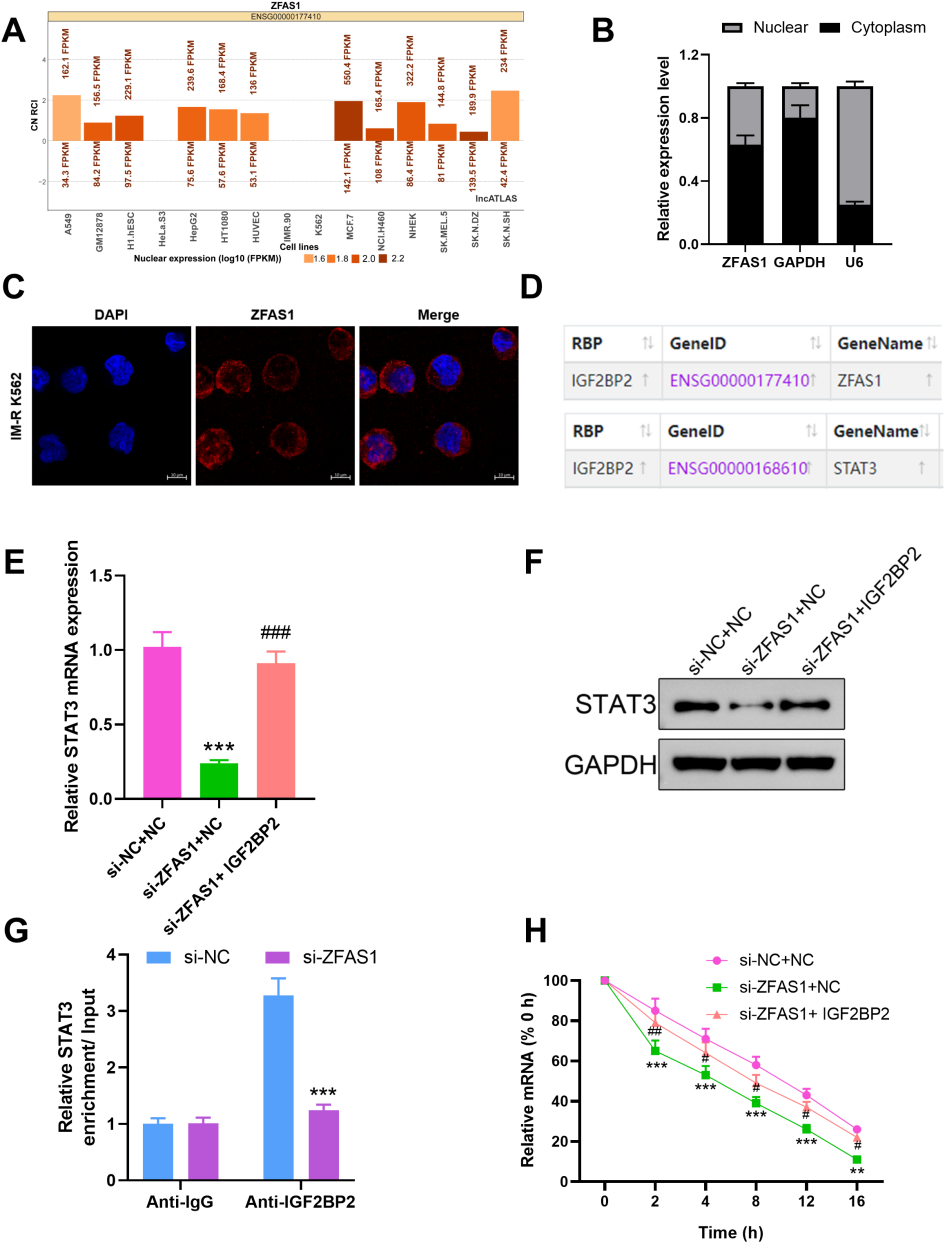
Mechanistic insights into *ZFAS1* regulation of glucose metabolism through *STAT3* and *IGF2BP2* in CML cells. **(A)** Prediction of *ZFAS1* localization using the LncATLAS database, indicating predominant cytoplasmic localization. **(B)** Cytoplasmic/nuclear RNA separation experiments confirming the cytoplasmic localization of *ZFAS1* in CML cells. **(C)** RNA FISH showed that *ZFAS1* located in the cytoplasm in cells, scale bars, 10 μm. **(D)** Bioinformatics prediction using starBase database showing potential interaction between *ZFAS1* and IGF2BP2. **(E, F)** Effect of *ZFAS1* knockdown on *STAT3* expression and the reversal of this effect by *IGF2BP2* overexpression. **(G)** RNA immunoprecipitation (RIP) analysis showing interaction between *ZFAS1*, *STAT3*, and IGF2BP2. Knockdown of *ZFAS1* reverses *STAT3* enrichment on IGF2BP2 protein. **(H)** Effect of *ZFAS1* knockdown on *STAT3* mRNA stability and the reversal of this effect by *IGF2BP2* overexpression. ^**^ *p*<0.01, ^***^ *p*<0.001 *vs*. si-NC or si-NC+NC; ^#^ *p*<0.05, ^##^ *p*<0.01, ^###^ *p*<0.001 *vs*. si-ZFAS1+NC.

Using the LncATLAS database, *ZFAS1* was predicted to be predominantly located in the cytoplasm of CML cells. This was further confirmed by cytoplasmic/nuclear RNA separation experiments, which showed that *ZFAS1* is mainly present in the cytoplasm (**Figures 4A, B**). RNA FISH was used to investigate the cellular localization of *ZFAS1* in IM-R K562 cells (**Figure 4C**). The results showed that *ZFAS1* was primarily localized in the cytoplasm of IM-R K562 cells. We hypothesized that *ZFAS1* may exert its function in IM-R K562 cells through post-transcriptional regulation. It has been suggested that lncRNAs may interact with RBPs to regulate their downstream target genes. IGF2BP2, a classical RBP, has been shown in multiple studies to play a role in regulating mRNA stability, thereby affecting tumor progression. Bioinformatic analysis using the starBase database predicted that *ZFAS1* might promote *STAT3* expression through the IGF2BP2 (**Figure 4D**). After *ZFAS1* silencing, *STAT3* mRNA expression was markedly suppressed in IM-R cells, with levels dropping from 1.00 ± 0.10 in the si-NC+NC group to 0.24 ± 0.02 in the si-ZFAS1+NC group (*p <*0.001). However, this effect was reversed by the overexpression of IGF2BP2, with levels increasing from 0.24 ± 0.02 in the si-ZFAS1+NC group to 0.91 ± 0.08 in the si-ZFAS1+ IGF2BP2 group (*p <*0.001), indicating that ZFAS1 mediates STAT3 expression through IGF2BP2 (**Figure 4E**). Western blot analysis further confirmed the protein expression changes of STAT3 after ZFAS1 knockdown and IGF2BP2 overexpression (**Figure 4F**), which is consistent with the mRNA expression trends shown in **Figure 4E**. Moreover, RIP experiments demonstrated that *ZFAS1* interacts with *STAT3*, and *STAT3* co-immunoprecipitates with IGF2BP2. *ZFAS1* knockdown significantly reduced IGF2BP2-mediated enrichment of *STAT3* mRNA, with levels dropping from 3.28± 0.3 in the si-NC group to 1.24± 0.10 in the si-ZFAS1 group (*p <*0.001, **Figure 4G**), confirming the interaction between *ZFAS1*, *STAT3*, and IGF2BP2. *ZFAS1* knockdown led to a decrease in the stability of *STAT3* mRNA (*p <*0.001), an effect that was reversed by the overexpression of IGF2BP2 (*p <*0.05, **Figure 4H**). This indicates that ZFAS1 enhances the stability of *STAT3* mRNA through IGF2BP2.These results elucidate the mechanism by which *ZFAS1* regulates glucose metabolism reprogramming in CML cells. By interacting with IGF2BP2, *ZFAS1* stabilizes *STAT3* mRNA, thereby promoting *STAT3* expression and contributing to IM-R.

### 3.4 STAT3 overexpression reverses the effects of ZFAS1 knockdown on imatinib sensitivity and glucose metabolism reprogramming in IM-R K562 cells

**Figure 5:**
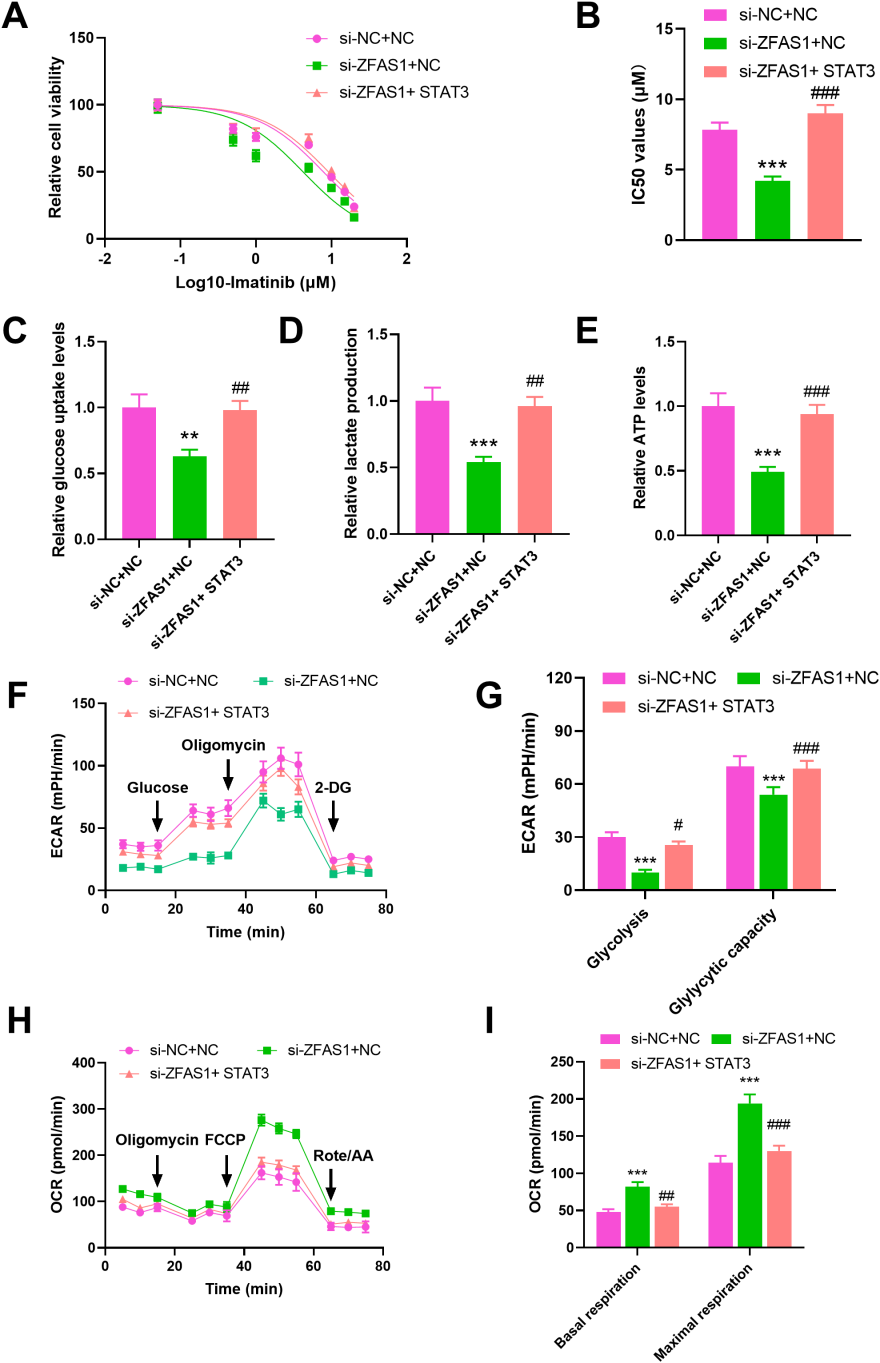
STAT3 overexpression reverses the effects of *ZFAS1* knockdown on imatinib sensitivity and glucose metabolism reprogramming in IM-resistant K562 cells, including si-NC+NC, si-ZFAS1+NC, and si-ZFAS1+STAT3 IM-R K562 cells. **(A)** Drug sensitivity assay by CCK-8 showing relative cell viability of cells treated with varying concentrations of IM. **(B)** IC50 values were determined via CCK-8 assay after 48 hours of imatinib treatment. **(C)** Relative glucose uptake levels. **(D)** Relative lactate production. **(E)** Relative ATP levels. **(F)** ECAR over time. **(G)** Glycolysis and glycolytic capacity. **(H)** OCR over time. **(I)** Summary of basal respiration and maximal respiration. ^**^ *p*<0.01, ^***^ *p*<0.001 *vs*. si-NC+NC; ^#^ *p*<0.05, ^##^ *p*<0.01, ^###^ *p*<0.001 *vs*. si-ZFAS1+NC.

To explore whether STAT3 mediates the regulatory effects of ZFAS1 on imatinib sensitivity and metabolism, we performed rescue experiments by overexpressing STAT3 in ZFAS1-knockdown cells. Drug sensitivity assays showed that knockdown of ZFAS1 significantly enhanced the sensitivity of IM-R K562 cells to imatinib, as indicated by a decrease in the IC50 value IC50 value from 7.839 µM (*p <*0.001) to 4.216 µM (*p <*0.001). Overexpression of *STAT3* reversed the increased sensitivity induced by *ZFAS1* knockdown, as indicated by a decrease in the IC50 value IC50 value from 4.216 µM to 9.149 µM (*p <*0.001, **Figures 5A, B**). *ZFAS1* knockdown resulted in a significant reduction in glucose uptake (0.63 ± 0.05), lactate production (0.54 ± 0.04), and ATP levels (0.49 ± 0.04) in IM-R K562 cells as compared to si-NC+NC group (1.00 ± 0.1). These effects on glucose uptake (0.98 ± 0.07), lactate production (0.96 ± 0.07), and ATP levels (0.94 ± 0.08) were reversed by the overexpression of *STAT3*, indicating that *STAT3* can mitigate the metabolic alterations caused by *ZFAS1* knockdown (*p <*0.001, **Figures 5C-E**). ECAR measurements showed that *ZFAS1* knockdown significantly inhibited both basal glycolysis (10 ± 1.6, *p <*0.001) and glycolytic capacity (54 ± 4.2, *p <*0.001), which was rescued by *STAT3* overexpression (25.67 ± 1.90; 68.67 ± 4.50, *p <*0.05/0.001, **Figures 5F, G**). OCR analysis showed that *ZFAS1* knockdown significantly increased both basal (82 ± 6.2, *p <*0.001) and maximal mitochondrial respiration (194 ± 12.1, *p <*0.001), whereas STAT3 overexpression reversed these effects (55 ± 3.5; 130 ± 7.2, *p <*0.01/0.001, **Figures 5H, I**), indicating a metabolic shift from oxidative phosphorylation back to glycolysis.

### 3.5 ZFAS1 induces HIF1α upregulation through STAT3

**Figure 6:**
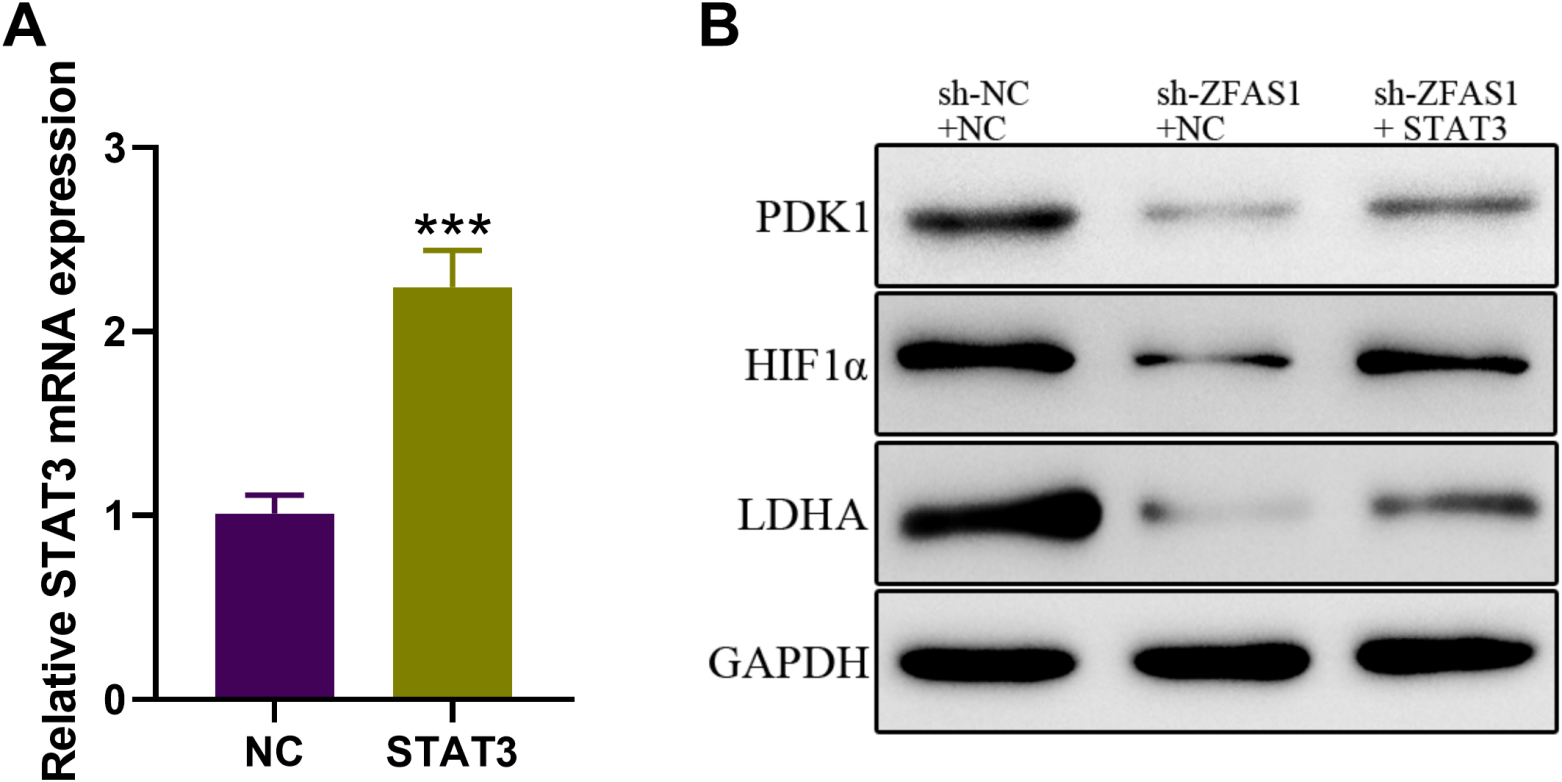
*ZFAS1* promotes HIF1α upregulation through *STAT3* in IM-resistant K562 cells. **(A)** Relative expression of *STAT3* in IM-R K562 cells transfected with *STAT3* plasmid compared to negative control (NC). **(B)** Western blot analysis showing the protein expression levels of HIF1α, LDHA, and PDK1 in si-NC, si-ZFAS1, and si-ZFAS1 + STAT3 transfected IM-R K562 cells. Data are presented as mean ± SD. ^***^ *p*<0.001 *vs*. NC.

To further confirm whether *STAT3* mediates the effects of *ZFAS1* on glycolytic activity, we overexpressed *STAT3* in *ZFAS1*-silenced CML cells. This approach aimed to determine if restoration of *STAT3* levels could rescue the metabolic suppression caused by ZFAS1 knockdown. In addition, we examined the expression of HIF1α, a key transcription factor involved in glycolysis (29), which is known to be transcriptionally regulated by *STAT3* (30). Therefore, we assessed whether *ZFAS1* influences the *STAT3–HIF1α* axis to regulate glycolysis in CML cells. We further assessed the protein levels of LDHA and PDK1, two well-established downstream targets of HIF1α. The expression level of *STAT3* mRNA was significantly increased in IM-R K562 cells transfected with *STAT3*-overexpression vector (2.24 ± 0.20) compared to the NC group (1.01 ± 0.10, *p <*0.001), demonstrating successful overexpression (**Figure 6A**). Western Blot analysis (**Figure 6B**) revealed that knockdown of *ZFAS1* introduced a reduction of HIF1α, LDHA, and PDK1 expression levels. However, the overexpression of *STAT3* reversed the *ZFAS1* knockdown effects, restoring the expression levels of these proteins. These findings indicate that *ZFAS1* promotes the upregulation of HIF1α through the activation of *STAT3*. The elevated levels of HIF1α and its downstream targets suggest enhanced glycolytic activity, which may contribute to the metabolic reprogramming and drug resistance observed in CML cells.

### 3.6 2-DG reverses the effect of STAT3 overexpression on imatinib sensitivity in IM-R K562 cells

**Figure 7:**
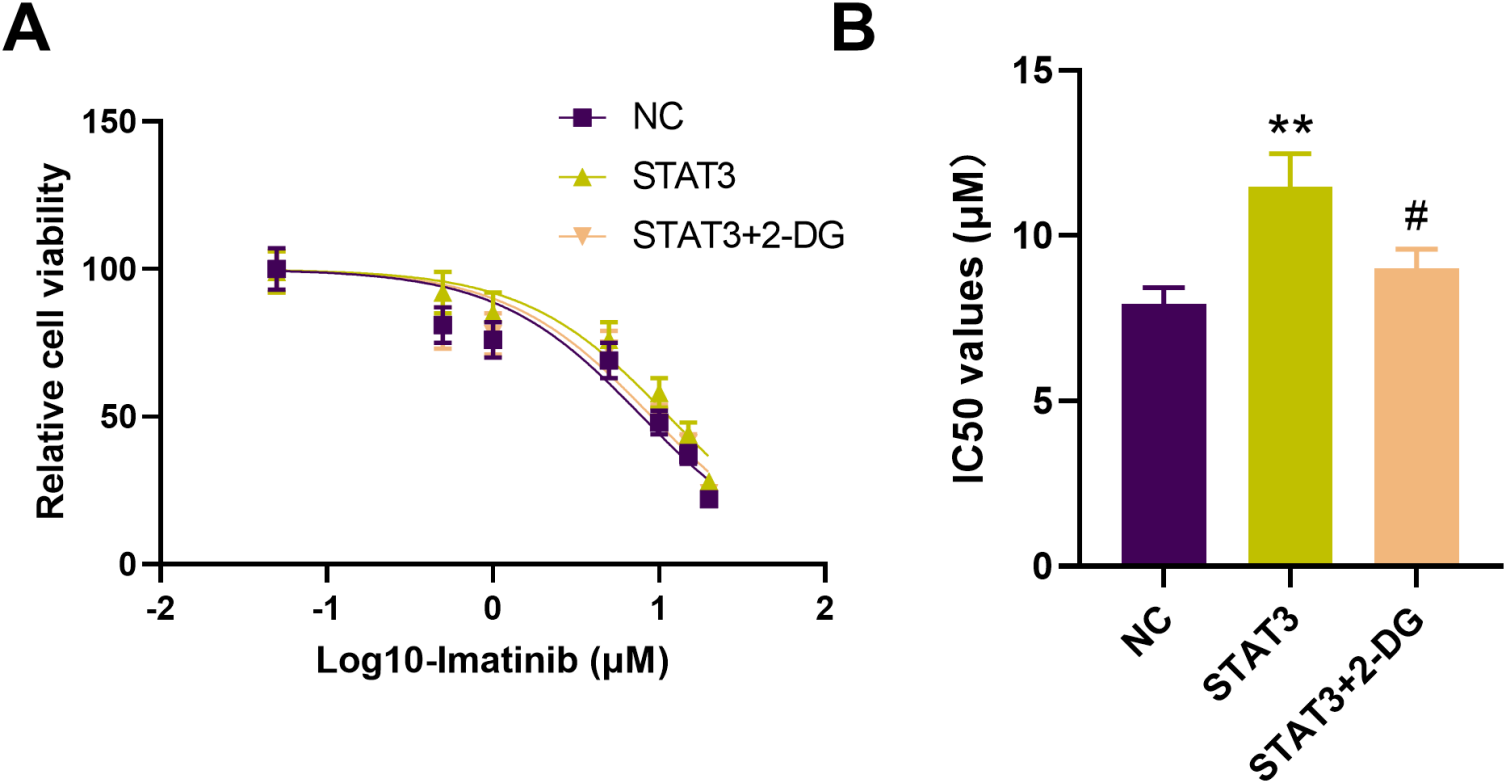
Effect of 2-DG on *STAT3*-mediated imatinib resistance in IM-R K562 cells. **(A)** CCK-8 assay shows relative cell viability of IM-R K562 cells with negative control (NC), *STAT3* overexpression (*STAT3*), and *STAT3* treated with 2-DG (*STAT3 +* 2-DG) under varying concentrations of imatinib. **(B)** IC50 values were determined via CCK-8 assay after 48 hours of imatinib treatment. ^**^ *p*<0.01 *vs*. NC; ^#^ *p*<0.05 *vs*. STAT3.

Drug sensitivity assays using CCK-8 showed that overexpression of *STAT3* significantly decreased the sensitivity of IM-R K562 cells to imatinib, as evidenced by an increased IC50 value. However, treatment with the glycolysis inhibitor 2-DG reversed the effect of *STAT3* overexpression, restoring imatinib sensitivity in these cells (**Figure 7A**). Specifically, the IC_50_ value for imatinib in STAT3-overexpressing cells (11.48 µM, *p <*0.01) was significantly higher compared to control cells (7.929 µM), but this increase was negated by 2-DG treatment (9.063 µM, *p <*0.05, **Figure 7B**). STAT3-mediated imatinib resistance in IM-R K562 cells is, at least in part, dependent on enhanced glycolytic activity. The reversal of this resistance by 2-DG highlights the potential therapeutic utility of targeting glycolysis in overcoming STAT3-induced drug resistance in CML.

## 4 Discussion

In this study, we demonstrate that the long noncoding RNA ZFAS1 exerts oncogenic effects in CML by promoting glucose metabolism and activating STAT3 signaling. Our findings align with prior reports highlighting ZFAS1’s involvement in other hematologic malignancies and solid tumors. Notably, ZFAS1 has been implicated in enhancing glycolysis and tumor proliferation via its interaction with key metabolic regulators.

Previously, it is highlighted the role of lncRNAs in cancer progression and drug resistance, but the specific involvement of *ZFAS1* in CML resistance mechanisms was not well understood before this study (31). The present study further demonstrated that knocking down *ZFAS1* expression using shRNA strongly increased the sensitivity of IM-R CML cells to imatinib treatment. These findings suggest that *ZFAS1* may be a potential therapeutic target for overcoming IM resistance in CML. Notably, the upregulation of *ZFAS1* has been extensively reported in various malignancies, where it contributes to tumor progression and metastasis (32). Moreover, *ZFAS1* has also been associated with drug resistance in multiple cancer types, such as melanoma, ovarian cancer, and hepatocellular carcinoma (32). The current findings regarding the involvement of *ZFAS1* in IM resistance in CML are consistent with these previous reports and further underscore the therapeutic potential of targeting *ZFAS1* in cancer treatment. In addition, knockdown of the *ZFAS1* has been shown to significantly alter the metabolic profile of cancer cells. In our study, *ZFAS1* knockdown in IM-R K562 cells induced a metabolic shift from glycolysis toward oxidative phosphorylation. These results provide new mechanistic insights into the anti-tumor effects of *ZFAS1* inhibition and reveal the metabolic adaptations associated with overcoming drug resistance.

A previous study demonstrated that compared to CML patients in chronic phase (CML-CP), the expression of *STAT3* in the bone marrow samples of CML patients in accelerated phase/blast phase (CML-AP/BP) was significantly elevated; additionally, the level of *STAT3* expression in IM-R K562/G01 cells was significantly higher than that in K562 cells (33). In our study, the expression of *STAT3* is significantly elevated in IM-R K562 cells. The study demonstrates a positive correlation between the expression of *STAT3* and *ZFAS1*. However, the potential interaction between *STAT3* and *ZFAS1* is still unclear. The selection of IGF2BP2 as a key interacting protein in this study is based on its well-established role as an RBP that regulates the stability and translation of specific mRNAs. IGF2BP2 has been implicated in the stabilization of mRNAs involved in critical cellular processes such as growth, differentiation, and metabolism, which are essential for cancer progression. Besides, many studies reported that lncRNAs has been reported to regulate downstream genes through interacting with IGF2BP2 (34–39). Furthermore, starBase database predicts that both *ZFAS1* and *STAT3* bind to IGF2BP2. *ZFAS1* was found to interact with IGF2BP2, enhancing the stability of *STAT3* mRNA and thereby promoting *STAT3* expression. The upregulation of *STAT3* by *ZFAS1* was shown to lead to the increased expression of *HIF1α*. The elevated HIF1α, in turn, resulted in the increased expression of the glycolytic enzymes LDHA and PDK1. The mechanism proposed in this study, where *ZFAS1* interacts with IGF2BP2 to stabilize *STAT3* mRNA and promote its expression, provides a novel insight into the regulation of *STAT3* in the context of CML drug resistance (40). Previous study has also highlighted the oncogenic role of *STAT3* in various cancers, where its constitutive activation is associated with poor prognosis and treatment resistance (41). The current findings build upon this by revealing the interplay between *ZFAS*1, *IGF2BP*2, and *STAT3*, which appears to be an important mechanism driving cancer progression. The regulation of HIF1α, LDHA, and PDK1 by the *ZFAS1-STAT3* axis is a novel insight that can have significant implications for understanding and targeting cancer metabolism.

Previous studies have also highlighted the role of *STAT3* activation in conferring resistance to *BCR::ABL1* inhibitors, including imatinib, in CML (42). Also, *STAT3*’s central role in mediating drug resistance in CML through metabolic reprogramming. For instance, Tezcanli Kaymaz et al. (43) reported that *STAT3* silencing sensitizes nilotinib-resistant CML cells by switching their energy metabolism from glycolysis to mitochondrial respiration. Our data further support this by demonstrating that inhibition of *ZFAS1* reduces *STAT3* expression and downregulates glycolytic enzyme expression, leading to diminished glucose uptake and lactic acid production. Moreover, our use of the glycolytic inhibitor 2-DG revealed that *ZFAS1*’s effects on CML cell survival are, at least in part, mediated by glycolysis. This is consistent with the observations of Patel et al. (44), who showed that *STAT3* promotes drug persistence in CML by shifting cellular metabolism toward glycolysis, thereby supporting leukemic stem cell survival. Interestingly, the role of *STAT3* in cancer is not restricted to CML. In lung cancer, Zheng et al. (45) demonstrated that *STAT3* inhibitors could sensitize resistant cells to EGFR-TKIs, further implicating *STAT3* as a universal driver of drug resistance via metabolic control. These cross-cancer observations support the broader relevance of our findings and the therapeutic potential of targeting the *ZFAS1–STAT3* axis.

There are still some research limitations in this study. This study primarily focused on *in vitro* experiments. *In vivo* studies are required to confirm the therapeutic potential of targeting the *ZFAS1/STAT3* axis in animal models and clinical settings. While the study provided insights into the interaction between *ZFAS1* and *STAT3*, further investigation is needed to fully elucidate the downstream signaling pathways and molecular mechanisms involved in glucose metabolism reprogramming.

While our study provides compelling evidence for the involvement of the *ZFAS1/STAT3* axis in regulating IM resistance in CML cells through reprogramming of glucose metabolism, the findings are limited by the exclusive use of *in vitro* models. Further studies, particularly in animal models, are needed to validate these findings and assess their physiological relevance in a whole-organism context. Such investigations will contribute to a more comprehensive understanding of the therapeutic potential of targeting the *ZFAS1/STAT3* pathway in CML. To facilitate the clinical translation of these findings, future studies should include well-designed clinical trials aimed at evaluating the efficacy of *ZFAS1* and *STAT3* as therapeutic targets for overcoming imatinib resistance. Beyond this, broader transcriptomic and signaling analyses should encompass additional lncRNAs and pathways that may interact with *ZFAS1* and *STAT3*, thereby elucidating the complexity of resistance mechanisms. Moreover, the exploration of combination therapies involving glycolysis inhibitors, like 2-DG, in conjunction with imatinib and other TKIs, should be pursued in both preclinical and clinical settings to develop more effective treatment strategies for patients with CML (46). Furthermore, long-term studies are needed to assess the sustained impact of targeting the *ZFAS1/STAT3* axis on CML progression, relapse rates, and overall patient survival.

Collectively, our study highlights a novel regulatory mechanism by which *ZFAS1* enhances *STAT3* activation and glycolysis in CML. This mechanism provides insight into how metabolic reprogramming supports TKI resistance and suggests that *ZFAS1* or *STAT3* could serve as therapeutic targets to overcome resistance and improve treatment outcomes.

## Data Availability

The raw data supporting the conclusions of this article will be made available by the authors, without undue reservation.
